# Effects of aqueous extracts of dried calyx of sour tea (*Hibiscus sabdariffa* L.) on polygenic dyslipidemia: A randomized clinical trial

**Published:** 2018

**Authors:** Majid Hajifaraji, Mohammad Matlabi, Farihe Ahmadzadeh-Sani, Yadollah Mehrabi, Mohammad Salem Rezaee, Homa Hajimehdipour, Abbas Hasanzadeh, Katayoun Roghani

**Affiliations:** 1 *National Nutrition and Food Technology Research Institute, Faculty of Nutrition & Food Technology Shahid Beheshti University of Medical Sciences, Iran*; 2 *Department of Health Education and Promotion, Gonabad University of Medical Sciences, Gonabad, Iran *; 3 *Food and Drug Deputy, Mashhad University of Medical Sciences, Iran *; 4 *Department of Epidemiology, College of Public Health Shahid Beheshti University of Medical Sciences, Iran *; 5 *Ebnesina Hospital, Mashhad University of Medical Sciences, Iran*; 6 *Department of Traditional Medicine Shahid Beheshti University of Medical Sciences, Iran*; 7 *Department of Humanities, Gonabad University of Medical Sciences, Iran*; 8 *Natural Products & medicinal plants Research Center, North Khorsan University of Medical Sciences, Bojnurd, Iran*

**Keywords:** Hibiscus sabdariffa L., Polygenic dyslipidemia, Sour tea, Lipid profile

## Abstract

**Objective:**

Dyslipidemia has been considered as a major risk factor for coronary heart disease. Alternative medicine has a significant role in treatment of dyslipidemia. There are controversial findings regarding the effects of sour tea on dyslipidemia. The aim of this study was to evaluate the impact of aqueous extract of dried calyx of sour tea on polygenic dyslipidemia.

**Materials and Methods:**

This clinical trial was done on 43 adults (30-60 years old) with polygenic dyslipidemia that were randomly assigned to the intervention and control groups. The control group was trained in lifestyle modifications at baseline. The intervention group was trained for lifestyle modifications at baseline and received two cups of sour tea daily, and both groups were followed up for 12 weeks. Lipid profile was evaluated at baseline, and six and 12 weeks following the intervention. In addition, dietary and physical activity assessed at baseline for twelve weeks.

**Results:**

Mean concentration of total cholesterol, HDL-C and LDL-C significantly decreased by up to 9.46%, 8.33%, and 9.80%, respectively, after 12 weeks in the intervention group in comparison to their baseline values. However, LDL-C/HDL-C ratio significantly increased by up to 3.15%, following 12 weeks in the control group in comparison to their baseline values. This study showed no difference in lipid profiles between the two groups, except for HDL-C concentrations.

**Conclusion:**

sour tea may have significant positive effects on lipid profile of polygenic dyslipidemia subjects and these effect might be attributed to its anthocyanins and inflation factor content. Therefore, sour tea intake with recommended dietary patterns and physical activity can be useful in regulation of lipid profile in patients with polygenic dyslipidemia.

## Introduction

Dyslipidemia is a major risk factor for coronary heart disease, which is the leading cause of mortality worldwide. Dyslipidemia treatment can reduce the risk of heart disease by up to 30% (Goff et al., 2006 ▶). The prevalence of hypercholesterolaemia in 19 countries of 3 continents varied across populations from 3% to 53% in men, and from 4% to 40% in women (Tolonen, 2005 ▶); the corresponding values in Iranian urban population were 72.3% and 65.8% among men and women, respectively (Azizi, 2002 ▶). Generally, dyslipidemia has been shown as low HDL-C among men and hypercholesterolaemia among women (Varady and Jones, 2005 ▶). The LDL-C/HDL-C ratio is a valuable and standard tool to evaluate CHD (Luz Fernandez, 2008 ▶; Pagana et al., 2009 ▶). 

Prospective researches indicated that dietary factors such as low intake of trans fat, high intake of whole grains, fruits and vegetables, and dairy products, and replacement of saturated fat or refined carbohydrates with unsaturated fats attribute to substantial reductions of cardiovascular risks (Mozaffarian, 2008 ▶). Several nutraceuticals such as antioxidant phytochemicals used in clinical trials had favorable effects on prevention and treatment of dyslipidemia (Alnoory, 2013 ▶, Derosa, 2013 ▶). Sour tea (*Hibiscus sabdariffa* L.) is a nutraceutical compound that contains mucilage, pectin, anthocyanins, polyphenols, hibiscus and citric acid, and helps to improve lipid profile (Ali, 2005 ▶; Lin, 2007 ▶; Mahadevan, 2009 ▶; Mozaffari and Khosravi, 2009 ▶; Gurrola. et al., 2010 ▶; Ramirez and Rodrigues, 2011 ▶; MaffoTazoho et al., 2011 ▶). *Hibiscus sabdariffa* L. (family Malvaceae) widely grows in tropical areas of Iran. Anthocyanins found in the calyx of sour tea have a variety of biological effects including improving blood pressure and lipid profile (Haji Faraji, 1999 ▶; Ajay, 2007 ▶). These effects have also been shown for polyphenols and hibiscus acid (Mozaffari and Khosravi, 2009 ▶; Ochani D'Mello, 2009 ▶; Sabzghabaee, 2013 ▶). Furthermore, anthocyanins have a protective effect on LDL-C against oxidation and atherosclerosis (Hopkins, 2013 ▶). Hirunpanich et al. showed that four-week treatment with sour tea resulted in 8.3-14.4% reduction of cholesterol levels in hypercholesterolemic rats. Furthermore, sour tea reduced triglycerides, LDL-C, and LDL-C/HDL-C ratio (Hirunpanich et al., 2006 ▶). 

The present study was designed to investigate the effect of aqueous extract of dried calyx of sour tea (*Hibiscus sabdariffa *L.) on the lipid profile of subjects with polygenic or nonfamilial dyslipidemia.

## Materials and Methods

A randomized controlled clinical trial was conducted comprising 43 adults (6 men and 37 women of 30-60 years old) with polygenic dyslipidemia, based on the criteria of Mosby's Diagnostic and Laboratory Test Reference (Pagana K.D., 2009 ▶). The participants were selected from 22-Bahman hospital, Gonabad, Iran in 2012. The recommended treatment for familial dyslipidemia was therapeutic lifestyle changes (TLCs) with lipid-lowering drugs; therefore, the subjects with polygenic dyslipidemia who were not using lipid-lowering drugs entered this study. The patients with polygenic dyslipidemia who had no history of familial dyslipidemia based on self-reports and had at least of the following criteria were included: a) total cholesterol > 200 mg/dl, b) LDL-C >180 mg/dl, c) HDL-C <45 mg/dl for men and <55 mg/dl for women, Other inclusion criteria were having no history of diseases like diabetes, cardiovascular diseases, nephritic syndrome, liver diseases, and thyroid gland dysfunction, not taking lipid-lowering drugs, not smoking, and not having special diets such as being vegetarian. The subjects who had allergy to sour tea, preferred not to drink sour tea, or took a trip during the course of study were excluded. Written informed consent was obtained from each patient. Ethics approval for this study was received from the Ethics Committee of the International Branch of Shahid Beheshti University of Medical Sciences (SBMU), Tehran, Iran.

Sample size was determined according to a previous study (Mozaffari and Khosravi, 2009 ▶) and the comparison of means formula and with regard to 5% type 1 error and 80% power, suitable sample size was calculated to be 25 subjects in each group. The subjects were randomly assigned into intervention and control groups. The participants in both groups were trained for lifestyle modifications, including diet and physical activity, at baseline they were asked to follow these instructions during the study period. Since both groups used black tea according to their regular diet, then, it was recommended to keep it during the study period. The 50 patients were divided into two control and intervention groups based on the said properties. In the first 6 weeks, 3 subjects were failed from intervention group and 4 subjects also were failed from the control group. But in the 12^th^ week only 1 subject was failed from each group. Then, after the 12^th^ week the data for 21 subjects from the intervention group and 20 subjects for the control group were analyzed. The intervention group received two cups of sour tea instead of two cups of black tea daily between meals, for 12 weeks. Sour tea was prepared as follows: one spoonful (2 grams) of pulverized sour tea was added to a cup of boiling water (240 ml) and left for 30 min at room temperature. The lipid profile was evaluated at baseline, and six and 12 weeks after the initiation of the trial. At each time point, samples were taken after 12 to 14 hr of fasting. 

Dorsa sour tea (Mohammad Ismail Vakili Company, Gonabad, Iran) was analyzed in the Traditional Medicine and Materia Medica Research Center of Shahid Beheshti University of Medical Sciences, Tehran, Iran. The amount of chemical components such as total anthocyanins and inflation factor was determined by standard laboratory methods (World Health Organization, 1998, British Pharmacopoeia Commission, 2009). Sour tea was approved for use if it met the following criteria: anthocyanins based on cyanidin 3-O-glucosidechloride 0.170±0.002g/100 g and an inflation factor of 5.5 per one gram of plant. The patients were given 150-gram packages of Dorsa sour tea monthly, and the process of tea preparation was mentioned on the package. Subjects were informed regarding the study protocol. Daily consumption of sour tea was confirmed by patients. When the next package was given to the subjects, the remaining of the previous package of sour tea was received and if sour tea was used by other family members, it was asked to be mentioned in the form of daily consumption. At baseline, 5 CC blood from the forearm vein was obtained from the subjects following 12-14 hr overnight fasting. The serum was separated for the measurement of total cholesterol (TC), triglycerides (TGs), high-density lipoprotein cholesterol (HDL-C), low-density lipoprotein cholesterol (LDL-C), and fasting plasma glucose (FPG). Dietary intake was evaluated by a valid and reliable food frequency questionnaire (Esfahani F., 2010, Asghari G., 2012) and analyzed by Nutritionist IV Diet Analysis software (version 3.5.2). Physical activity was assessed using the MET questionnaire (Guidelines for Data Processing and Analysis of the International Physical Activity Questionnaire (IPAQ), 2005). At baseline, subjects in both groups were advised to consume whole grains, fruits and vegetables, low-fat dairy products, low-fat meat, fish, unsaturated fat instead of saturated fat, limited amount of trans fat, and 30 min of brisk walking on most days of the week. 

Fasting blood samples were taken following 12–14 hr overnight fasting at baseline, and six and 12 weeks after initiation of the trial. The analysis of samples was performed using autoanalyzer (Bio Tecnica -3000). Fasting plasma glucose, TC and TGs were measured by enzymatic methods using glucose oxidase, cholesterol oxidase and glycerol oxidase kits (Pars azmoon, Iran), respectively. LDL-C and HDL-C were measured by an enzymatic method using commercially available kits (Pars azmoon, Iran). 

**Statistical analysis**

Statistical analysis was performed using SPSS (version 18.0, SPSS Inc., Chicago, IL, USA). Data were presented as mean *± *SD. At baseline, Student’s T-test was used to compare quantitative variables and chi-square test was used to compare qualitative variables between the two groups. The repeated measures test was used to determine significant changes in lipid profile within each group, and analysis of covariance was used to determine significant changes in lipid profile between the two groups. In analysis, confounding variables such as age, body mass index, and physical activity were adjusted. p values <0.05 were considered statistically significant.

## Results

Mean values of baseline characteristics of patients of both groups are presented in Table 1. No significant differences in age, BMI, duration of disease, TGs, HDL-C, and LDL-C/HDL-C ratio, were observed between the two groups. However, significant differences were seen in physical activity (p=0.01), cholesterol (p=0.04), and LDL-C (p=0.04).

At baseline, based on the therapeutic lifestyle change (TLC) in diet components, no significant differences were seen between the two groups for daily nutrient intake; however, mean daily intakes of carbohydrates, fat, and saturated fatty acids were higher than the recommended limits in the control group, and mean intakes of carbohydrates, fat, saturated fatty acids and cholesterol were higher than the recommended limits in the intervention group (Table 2). At the end of the study, regarding the effect of foods (hydrogenated oil, animal oil, liquid oil, high-fat dairy, high-fat meat, and sweets) on lipid profile, no significant differences were seen between the two groups except for sweets (Table 3). Intervention and control groups differed in terms of physical activity at baseline (p=0.03), not at the end of the study (p=0.06).

**Table 1:** Comparison of quantitative variables between two groups before initiation of the trial.

**Variables**	**Control (n=22)** **(3 men and 19 women)**	**Intervention (n=21)** **(3 men and 18 women)**	**p-value**
**Age (years)**	49.04±8.26	47.76± 8.09	0.61
**Body mass index (kg/m** ^2^ **)**	27.80±4.21	27.61±4.14	0.88
**Duration of illness (months)**	27.27±36.47	29.24±37.05	0.86
**Physical activity (MET-min/week)**	1504.5±1339.9	723.7± 384.4٭	0.01
**Cholesterol (mg/dl)**	229.0±5.2	246.4±7.1٭	0.04
**Triglycerides (mg/dl)**	181.0±13.9	188.0±13.2	0.80
**HDL-C (mg/dl)**	43.4±1.7	43.8±1.29	0.75
**LDL-C (mg/dl)**	148.9±4.9	165.2±6.2٭	0.04
**LDL-C/HDL-C ratio**	3.49±.12	3.82±.17	0.13

* p≤0.05

**Table 2:** Comparison of mean and standard deviation values of daily nutrient intake between the two groups at baseline.

**Variables**	**Control (n=22)**	**Intervention (n=21)**	**p-value**
**Energy(Kcal)**	2106.7±587.9	2123.5±583.5	0.92
**Protein(g)**	56.25±19.51	56.75±17.14	0.93
**Carbohydrate(g)**	336.31±125.58	330.73±100.25	0.87
**Fat(g)**	69.59±14.61	73.48±23.85	0.52
**Cholesterol(mg)**	198.75±64.14	207.21±104.79	0.75
**Saturated fatty acid(mg)**	22.59±6.33	26.87±10.20	0.10
**Dietary fiber(g)**	27.77±11.78	25.47±9.24	0.48
**Protein(Percent)**	10.18±1.53	10.43±2.20	0.67
**Carbohydrate(Percent)**	60.23±6.46	59.62±5.85	0.52
**Fat(Percent)**	29.54±6.81	29.81±5.30	0.89

The effects of sour tea on lipid profile before and after intervention are presented in Table 4. The repeated measures test showed no significant difference in TC (p=0.35), TGs (p=0.56), LDL-C (p=0.29), and LDL-C/HDL-C ratio (p=0.11) between the two groups; however, HDL-C was significantly different (p<0.002) between the two groups. Although no clinically significant differences in lipid profiles were observed between the two groups, but this study showed significant reduction in TC (p=0.03), LDL-C (p=0.03), HDL-C (p<0.001) in the intervention group. Our intervention did not induce significant changes in TGs (p=0.31) or, LDL-C/HDL-C ratio (p=0.39).

As presented in Table 4, there were significant differences in TC and LDL-C between two groups at baseline, but no significant differences were found in TGs, HDL-C, and LDL-C/HDL-C ratio. After six and 12 weeks, no significant differences were found in TC, TGs, LDL-C, and LDL-C/HDL-C ratio, between the two groups.

**Table 3:** Consumption of food in two groups before and after intervention

**p-value** 1	**12th Week**			**Baseline**			**variables**
	**p-value**	** Intervention (n=20)Number** ** (Percent)**	**Control (n=21)** **Number** ** (Percent)**	**p-value**	** Intervention (n=21)** **Number (Percent)**	**Control (n=22)** **Number** ** (Percent)**	
0.87	0.87	(50) 10	(52.4) 11	.870	(47.6) 10	(50) 11	Hydrogenated oil
0.2	0.73	(90) 18	(85.7) 18	.730	(85.7) 18	(81.8) 18	Animal oil
.860	.090	(55) 11	(80.9) 17	.170	(47.6) 10	(68.2) 15	Liquid oil
0.4	0.66	(65) 13	(71.4) 15	.440	(66.7) 14	(77.3) 17	High-fat dairy
0.68	0.02	٭(65) 13	(28.6) 6	.030	٭(81) 17	(50) 11	Sweets
0.31	0.06	(90) 18	(47.6) 10	.030	٭(85.7) 18	(54.5) 12	High-fat meat

1 before and after intervention.

**Table 4:** Mean and Standard Deviation Values of Lipid Profile during 12 Weeks of Intervention

**Variables**	**Control (n=22)**	**Intervention (n=21)**	**p-value**
**Total cholesterol (mg/dl)**			
**Baseline **	229.0±5.2	246.4±7.1٭	0.04
**Sixth Week**	218.1±5.6	229.5±8.2	0.23
**Twelfth Week (n=21 and 20)**	223.6±5.8	223.1 ±9.6	0.96
**Percent coefficient of variation**	-2.35	-9.46٭	
**p-value**	0.50	0.03	
**HDL-C (mg/dl)**			
**Baseline **	43.4±1.7	43.8±1.29	0.75
**Sixth Week**	43.6±1.5	41.8±1.4	0.54
**Twelfth Week (n=21 and 20)**	42.1±1.5	40.1 ±1.5	0.35
**Percent coefficient of variation**	-2.97	-8.33٭	
**p-value**	0.27	<0.001	
**LDL-C (mg/dl)**			
**Baseline **	148.9±4.9	165.2±6.2٭	0.04
**Sixth Week**	142.3±4.9	150.6±7.3	0.23
**Twelfth Week (n=21 and 20)**	148.9±5.0	149.0 ±9.1	0.96
**Percent coefficient of variation**	-1.95	-9.80٭	
***P*** **-value**	0.90	0.03	
**LDL-C/HDL-C ratio**			
**Baseline **	3.49±.12	3.82±.17	0.13
**Sixth Week**	3.30±.12	3.65±.20	0.2
**Twelfth Week (n=21 and 20)**	3.60±.16	3.76±.23	0.57
**Percent coefficient of variation**	+3.15٭	-1.57	
**p-value**	0.02	0.39	
**Triglycerides (mg/dl)**			
**Baseline **	181.0±13.9	188.0±13.2	0.80
**Sixth Week**	160.4±15.2	185.1±18.5	0.30
**Twelfth Week (n=21 and 20)**	158.9±14.4	169.4±17.9	0.65
**Percent coefficient of variation**	-12.26	-9.90	
**p-value**	0.11	0.31	

٭ p≤.05

**Figure 1:**
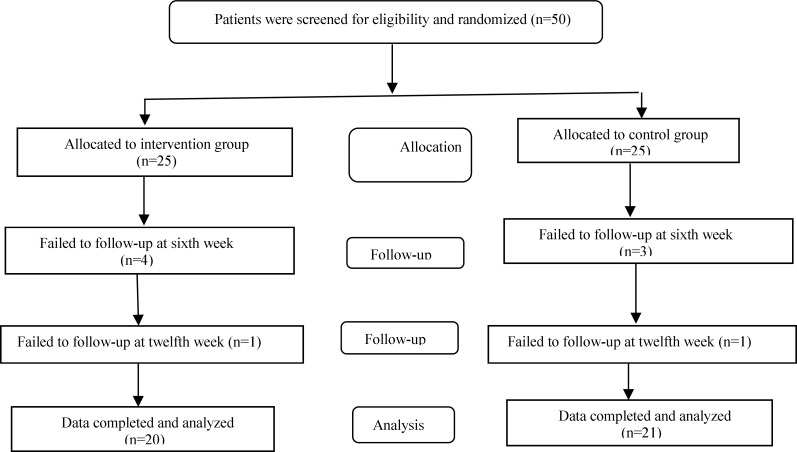
Patients Allocation, Follow-up and Data Analysis Diagram of the Study

## Discussion

In the present study, consuming aqueous extract of dried calyx of sour tea for 12 weeks caused 9.46, 9.80, 8.33, 1.57, and 9.90% reductions in TC, LDL-C, HDL-C, LDL-C/HDL-C ratio, and TGs, respectively, in the intervention group. Regarding the significant baseline differences in TC and LDL-C, due to loss of patients during the study, it is probable that no significant differences would be seen between the two groups at the end of the study. Lack of regulation of cholesterol levels may lead to serious pathological conditions. Cholesterol, particularly LDL-C, plays an important role in the pathogenesis of atherosclerosis.

As physical activity and dietary behaviors could be critical confounders, in this study, these variables were controlled; both case and control groups were treated similarly for physical activity and dietary pattern. The study showed statistically no significant difference regarding physical activity and food intakes (except for sweets) between two study groups. Therefore, consistent with previous studies, the overall decrease of plasma lipid levels in the intervention group, which was also mentioned previously, might be attributed to sour tea effects (Lin, 2007 ▶; Mozaffari and Khosravi, 2009 ▶; Gurrola and Diaz, 2010 ▶; MaffoTazoho, 2011 ▶). Lin et al, showed that four weeks of treatment with* H. sabdariffa *extract reduced serum cholesterol in men and women by about 1.85% (Lin, 2007 ▶). Also, Mozaffari-Khosravi et al. showed sour tea consumption for four weeks reduced TC, LDL-C, and TGs up to 7.6, 8, and 14.9%, respectively (Mozaffari and Khosravi, 2009 ▶). Diaz et al. showed that consumption of *H. sabdariffa *extract powder for one month, reduced TC levels by 2.52% and reduced TGs by 23.11%, in patients who had metabolic syndrome (Gurrola and Diaz, 2010 ▶). Tazoho et al. showed that drinking "foléré" (calyx of sour tea) by healthy men for nine days reduced the mean serum TC by 6.49% and the mean HDL-C by 19.70% compared to the baseline values (MaffoTazoho, 2011 ▶). Hirunpanich et al, showed that treatment of hypercholesterolemic rats with 500 and 1000 mg/kg aqueous extract of dried calyx of *H. sabdariffa* L. for six weeks reduced TC levels by 22 and 26%, respectively and also reduced LDL-C levels (Hirunpanich, 2006 ▶). In contrast, Mohagheghi et al., revealed that consumption of sour tea and black tea by hypertensive patients for 15 days, caused an upward trend in TC, HDL-C, LDL-C, and TGs in both groups (Mohagheghi et al., 2011 ▶). Moreover, the response to administration of natural compounds could be heterogeneous depending on the concentration, form, and dosage of the compound, duration of treatment, and the elaboration and chemical composition of the extract, physiological conditions, sex, age, hormonal status, and dietary intakes.

Based on our findings, some subjects did not completely adhere to the recommended healthy diet, which may explain why sour tea was not effective in all subjects.

Several clinical trials and *in vivo* studies have demonstrated a lipid-lowering effect for *H. sabdariffa *extract which was probably due to the effect of anthocyanins, one of the extract's major components (Lin, 2007 ▶; Mozaffari and Khosravi,2009 ▶) as well as the antioxidant properties and soluble fiber content of the sour tea (Sayago and Ayerdi, 2007 ▶).

 In this study, no significant side effects were reported. The major limitations of the current study were not using appropriate placebo, baseline difference between two groups in terms of TC and LDL-C. The strengths of the current study were using high quality sour tea; some studies have not reported constituents of the extracts.

In the current study, TC and LDL-C were significantly reduced in the intervention group during the course of the study. It is concluded that sour tea may have significant positive effects on lipid profile of subjects with polygenic dyslipidemia which were attributed to the presence of anthocyanins and inflation factor content. HDL-C significantly differed between the two groups following 12 weeks of treatment with sour tea. Total cholesterol, LDL-C, TGs, and LDL-C/HDL-C ratio did not differ between the two groups. Therefore, it is concluded that sour tea intake with recommended dietary patterns and physical activity can be useful in regulation of lipid profile in patients with polygenic dyslipidemia.
